# 수술 후 당뇨병 환자의 수액 주입 팔과 비주입 팔 간 손가락 끝 혈당 수치 비교: 단면 연구

**DOI:** 10.7586/jkbns.25.034

**Published:** 2025-08-22

**Authors:** MinJi Lee, SinJung Jung, MinJi Kim, MiRa Lee, KwonJu Moon, EunMi Kang, JiWoo Park, YeonJi Cho, GiBum Jin, SeungJoo Kim, YeZi Woo, JaHyun Kang

**Affiliations:** 1 Department of Nursing, Seoul National University Bundang Hospital, Seongnam, Korea; 1 분당서울대학교병원 간호본부; 2 College of Nursing • Research Institute of Nursing Science, Seoul National University, Seoul, Korea; 2 서울대학교 간호대학 • 간호과학연구소

**Keywords:** Diabetes mellitus, Postoperative care, Blood glucose, Blood glucose self-monitoring, Infusions, intravenous, 당뇨병, 수술 후 간호, 혈당, 자가 혈당 측정, 정맥 내 주입

## Abstract

**Purpose:**

This study investigated the effect of intravenous maintenance fluid infusion on blood glucose levels by comparing fingertip glucose measurements between the infused and non-infused arms of patients with diabetes in a recovery room after surgeries under general anesthesia.

**Methods:**

We initially included 151 patients with diabetes who were admitted to the recovery room of Seoul National University Bundang Hospital between June 4 and October 25, 2024. Sixteen patients were excluded because they received fluids that were hypothesized to potentially affect blood glucose levels during surgery, and seven were excluded due to notable blood glucose measurement errors, leaving 128 patients in the final analysis. The primary maintenance fluid was administered through one arm, whereas the other arm remained non-infused. Blood glucose levels were measured twice in both arms, using the same glucometer, and the average values were calculated. Data were analyzed using SPSS version 26.0, and the paired Student’s t-test was conducted to assess differences in fingertip glucose levels between the two arms.

**Results:**

Fingertip glucose levels exhibited a statistically significant difference between the infused and non-infused arms (t = –3.65, *p* < .001). The mean glucose level in the infused arms was 143.82 ± 36.44 mg/dL, which was lower than that in the non-infused ones (146.17 ± 37.46 mg/dL).

**Conclusion:**

Intravenous fluid infusion may affect peripheral blood glucose levels. This study highlights the importance of selecting appropriate sites for blood glucose monitoring in clinical settings, and provides evidence supporting accurate blood glucose monitoring and management in patients with diabetes.

## 서론

### 1. 연구의 필요성

당뇨병은 만성적인 고혈당증이 주요 소견인 대사 장애의 총칭으로[1], 기대 수명의 연장 및 생활양식의 변화로 전 세계적으로 유병률과 발병률이 빠르게 증가하여 장애 및 사망률을 초래하는 주요 보건 문제가 되었다[2,3]. 대한당뇨병협회에서 의료 빅데이터를 통해 조사한 통계[4]에 의하면, 2022년 우리나라 30세 이상 당뇨병 환자는 약 533만명으로 성인 약 7명 중 1명(14.8%)에 이르고, 30세 이상 약 10명 중 4명(41.1%)이 당뇨병 전 단계에 해당한다. 또한, 최근 11년간 우리나라 성인 당뇨병 환자는 11.8%에서 14.8%로 점차 증가하는 양상이며[4], 당뇨병 환자의 증가와 함께 당뇨병 환자가 수술을 받게 되는 빈도도 증가하게 되었다[2,5].

당뇨병 환자는 입원 중 신체 활동의 제한과 스트레스 호르몬 분비의 증가로 인해 인슐린의 요구가 더욱 높아지고[6], 비당뇨병 환자에 비해 수술 후 위 마비, 심혈관계 부작용, 신장 합병증, 감염 및 패혈증, 나아가 사망률의 위험이 증가한다[2,5]. 이와 같이, 당뇨병 환자의 수술 전후 혈당 관리는 매우 중요하므로 세심하고 집중적인 관리가 필요하며, 수술 후 회복실에 입실하면 반드시 혈당을 측정해야 한다[5,7,8].

이때, 당뇨 관리의 표준이 되는 전통적인 혈당 측정 방법은 손가락 끝에서 모세혈관혈을 채혈하여 혈당을 측정하는 자가 혈당 검사이다[9-11]. 이 방법은 임상적으로 공인되었으며, 편리하고 비용 효율적일 뿐만 아니라, 응급 상황이나 빈번한 혈당 측정이 필요한 경우에도 신속한 결과를 제공하여 환자 진료에 중요한 역할을 한다[9,11-13].

수술 환자에 있어 수술 전 또는 수술 중 혈액량의 감소는 이환율과 사망률의 주요 원인이므로, 조직액의 회복과 유지, 전해질의 항상성 및 체내 순환 혈량 유지를 위한 최적의 수액 요법은 수술 중 환자 관리에 필수적이다[14-16]. 이때 수술용 일차 유지 수액으로는 사람 혈장과 비교적 동일한 등장성의 균형 잡힌 정질용액(crystalloid)이 선택되며, 여기에는 Lactated Ringer's, Hartmann's, Plasma Lyte A 등이 포함된다[14].

이러한 수액 요법을 적용 중인 당뇨병 환자에게 손가락 끝에서 혈당을 측정할 때, 수액이 혈관에 주입되면 해당 손가락 끝 혈당 측정 결과에 영향을 줄 것으로 생각하여 수액을 주입하지 않는 쪽 손가락에서만 혈당을 반복적으로 측정하는 문제가 임상 현장에서 발생한다[6]. 하지만, 손가락 끝을 찌르는 방법은 인슐린 펜으로 주사하는 것보다 통증이 더욱 심하고[17], 손가락에는 통증 감지세포가 집중되어 있어 손가락 끝 동일 부위를 반복 검사하는 경우 심한 통증이 유발되어 혈당검사를 기피하여 혈당 조절에 실패하거나, 채혈 부위의 손상이 문제가 될 수 있다[11]. 또한, 반복적인 채혈로 인해 굳은살이 생기면 혈액 방울의 크기가 줄어들고, 한 번의 판독을 위해 여러 번 채혈을 시도해야 하는 상황이 발생할 수 있다. 이러한 불편을 해소하기 위해 손가락이 아닌 다른 부위에서 혈당을 측정하는 방법이 도입되고 있으나[18] 발가락에서 검사하는 경우는 당뇨병으로 인한 족부 질환의 발생 위험이 있고[6], 팔뚝에서 검사를 하는 경우 모세혈관 흐름과 밀도 감소로 혈당 변화 감지가 지연되어 저혈당증이 있거나 저혈당증 인식이 어려운 환자에게는 적절하지 않다[19].

정맥 내 혈류는 단순한 혈압 차이 외에도 정맥 주변 근육의 펌프 작용과 정맥 내 판막의 역류 방지 기전에 의해 항상 일정한 방향으로 흐른다[20]. 이러한 생리적 특성을 고려할 때, 정맥 주입된 수액이 카테터 삽입 부위보다 말단에 위치한 손가락 끝의 혈당 수치에 실제로 영향을 미치는지에 대한 과학적 근거는 부족하다. 즉, 수액이 주입되는 팔과 그렇지 않은 팔의 손가락 끝에서 측정된 혈당 수치 간의 차이에 대한 근거가 부족하므로, 이를 규명하기 위한 추가 연구가 필요하다.

선행 연구에 따르면, 당뇨병 환자와 비당뇨병 환자 모두에서 포도당 수액을 주입하는 팔과 주입하지 않는 팔의 손가락 끝 혈당 수치에 유의한 차이가 없다는 보고[6]가 있으나, 수술 후 마취 회복 환자를 대상으로 포도당이 아닌 수술 목적의 유지 수액을 주입하는 상황에서 양팔의 손끝 혈당 수치를 비교한 연구는 현재까지 보고된 바 없다.

이에 본 연구에서는 전신마취 하 수술 후 마취 회복 중인 당뇨병 환자를 대상으로 수술 중 사용하는 일차 유지 수액을 주입하는 팔과 주입하지 않는 팔의 손가락 끝 혈당 수치 비교를 통해 수액 주입이 혈당 수치에 영향을 미치는지 확인하여 자가 혈당 측정 시 임상간호 중재의 근거를 확립하고자 하였다.

### 2. 연구의 목적

전신마취 하 수술 후 마취회복실에 입실하여 수액 요법을 받고 있는 당뇨병 환자를 대상으로, 수액을 주입하고 있는 팔과 주입하지 않는 팔의 손가락 끝에서 각각 2회의 혈당 수치를 측정한 후 평균치를 비교하여 수액 주입이 혈당 수치에 영향을 미치는지 확인하기 위함이다.

### 3. 연구의 가설

수액을 주입하고 있는 팔과 주입하지 않는 팔의 손가락 끝 혈당 수치 간에 차이가 있을 것이다.

## 연구 방법

### 1. 연구 설계

본 연구는 당뇨병 환자에게 주입되는 수액이 손가락 끝 혈당 수치에 영향을 주는지 확인하기 위해 전신마취 하 수술 후 마취회복실에 입실한 환자에서, 수액을 주입하고 있는 팔과 주입하지 않는 팔의 손가락 끝 혈당 수치를 비교한 단면 연구이다.

### 2. 연구 대상

본 연구의 대상자는 경기도 소재 분당서울대학교병원에서 전신마취 하 수술을 받는 환자 중 당뇨병을 진단받은 환자로, 연구 목적과 절차에 관하여 충분한 설명을 제공받은 후 자발적으로 참여에 동의한 자를 대상으로 하였다.

표본 수는 G*Power 3.1.9.4 프로그램을 이용하여 paired t-test, 효과크기 0.25, 유의수준 .05, 검정력 .80을 기준으로 산정한 결과, 최소 128명이 필요한 것으로 나타났다. 이는 선행연구에서 효과크기가 명확히 보고되지 않았기 때문에, 일반적으로 널리 활용되는 Cohen[21]의 중간 수준 효과크기인 0.25를 적용하여 산출한 것이다. 이에 따라 최소 필요 인원인 128명에 탈락률 15%를 고려하여 총 151명을 모집하였고, 대상자 중 수술 중 혈당 수치에 영향을 줄 수 있는 수액을 투여한 16명과 측정값의 오류로 인한 7명을 제외하여 총 128명이 최종 분석에 포함되었다.

본 연구의 대상자는 만 19세 이상 성인으로 전신마취 수술 후 마취회복실에 입실하는 환자, 당뇨병을 진단받은 환자, 미국마취과학회(American Society of Anesthesiologists, ASA) 신체등급 Ⅲ미만인 환자, 한쪽 팔에서 수액 요법을 적용 중인 환자를 포함하였다. 반면, 양쪽 팔 또는 손에서 수액 요법을 적용 중인 환자, 상지 채혈 금지 환자(동정맥루 수술 환자이거나 동정맥루를 유지 중인 환자, 유방 수술 환자이거나 수술로 인해 arm save를 유지 중인 환자, 상지 수술 환자, 손 화상 환자), 말초신경병증 또는 말초혈관질환이 있는 환자, 응급 수술 환자, 혈당 수치에 영향을 줄 수 있는 약물(corticosteroids) 및 수액(dextrose)을 투여한 환자는 제외하였다.

### 3. 연구 도구

#### 1) 증례기록지

전자의무기록을 통해 대상자의 일반적 특성과 수술 관련 정보를 조사하였으며, 회복실 입실 후 수액을 주입하지 않는 팔과 주입하는 팔의 손가락 끝을 각각 한 번만 찔러 흘러나온 혈액으로 혈당을 두 차례 연속 측정하였고, 측정 결과는 증례 기록지에 기록하였다.

#### 2) 혈당 수치

혈당 측정은 자가 혈당 측정기(Barozen H Expert Plus, Handok, Seoul, Korea)를 사용하여 양쪽 손가락 끝에서 측정하였고, 2회 연속 측정 후 평균값을 이용하였다. 측정도구의 정확성과 일관성을 유지하기 위해 1개의 혈당 측정기를 사용하였다. 해당 기기는 국제표준화기구(International Organization for Standardization, ISO)의 기준[22]을 기반으로 한 산업통상자원부의 체외진단 시험시스템 요구 규격을 통과하였으며, 혈당 수치 측정 범위는 20~600 mg/dL이고 측정에 필요한 최소 검체량은 0.4 uL이었다. 매일 부서 내에서 대조시험(control test)을 통해 정도 관리를 실시하고 기기 이상 및 정상작동 여부를 확인하였다. 측정에 사용되는 시험지(strip)는 습기와 직사광선을 피해 보관하고 유효기간 내에 있음을 확인한 후 사용하였고, 채혈기구는 말초혈액을 얻기 위해 28 gauge의 1.8mm 일회용 란셋을 이용하였다.

### 4. 자료 수집

본 연구는 경기도 소재의 분당서울대학교병원에서 Institutional Review Board (IRB) 승인(B-2406-905-301)을 받은 후 2024년 6월 4일부터 2024년 10월 25일까지 자료 수집을 하였다.

#### 1) 측정자 교육

측정자 간의 편차를 최소화하기 위해 마취회복실에서 7년 이상의 경력을 가진 간호사 4명을 측정자로 선정하였으며, 상호 시범 과정에서 측정자 간의 일치율은 ISO에서 제시하는 혈당 측정기의 허용 오차 범위(± 15%) 내에서 100%를 보였다. 또한 측정의 신뢰도를 확보하기 위해 모든 측정자에게 교육을 실시하였으며, 이 교육에는 란셋 및 혈당 측정기의 표준 사용 방법, 동일한 혈액을 이용한 2회 연속 측정 방법, 수액 비주입 팔과 주입 팔의 혈액 채취 순서 및 시간 간격 등을 포함하였다.

#### 2) 자료 수집 절차

대상자의 일반적인 특성과 수술 관련 정보는 전자의무기록을 활용하여 수집하였다. 혈당 측정을 위한 진행 절차는 다음과 같다. 혈당 측정값의 반복 측정을 통해 신뢰도를 확보하고 기기 오차 가능성을 줄이기 위해 대상자가 마취회복실에 입실하면 수액을 주입하지 않는 팔의 검지손가락 끝을 한 번만 찔러 흘러나온 혈액으로 혈당을 두 차례 연속 측정하였다. 다음으로 수액을 주입하는 반대쪽 팔의 손가락 끝에서도 동일한 방법으로 혈당을 두 차례 연속 측정하였고, 두 측정값의 평균을 해당 부위의 최종 혈당 수치로 기록하였다. 그리고 기기 오차 가능성을 줄이기 위해 동일한 혈당 측정기 1대만을 사용하였고, 모든 대상자의 혈당 수치를 측정하는 순서는 수액을 주입하지 않는 팔에서 주입하는 팔의 순서로 동일하게 적용하였다. 연구에 사용된 혈당 측정기(Barozen H Expert Plus, Handok, Seoul, Korea)의 혈당 검사 측정시간은 5초 내외이므로[23] 반복 측정을 위한 시험지(strip) 교체 시간을 고려하여 한쪽 팔의 손가락과 다른 팔의 손가락 끝 혈당 측정 시까지 걸리는 시간은 2분을 넘지 않도록 하여 시간 경과에 따른 생리적 변화의 영향을 통제하였다. 또한 다른 요인의 영향을 최소화하기 위해 양손의 동일한 위치에 해당하는 손가락 끝을 채혈 부위로 지정하였다. 이후 양쪽 손가락 끝에서 2회 측정된 혈당 수치의 평균값 간 차이를 비교하였다.

### 5. 자료 분석

수집한 자료는 SPSS version 26.0 (IBM Corp., Armonk, NY, USA)을 이용하여 기술적 통계 분석을 사용하였다. 대상자의 일반적 특성과 수술 관련 특성은 빈도와 백분율, 평균과 표준 편차를 구하였고, 수액을 주입하고 있는 팔의 손가락과 주입하지 않는 팔의 손가락 끝 혈당 수치의 차이는 정규성 검정 후 paired t-test로 분석하였다.

### 6. 윤리적 고려

자료 수집 전 분당서울대학교병원의 IRB에 본 연구에 대한 계획서를 제출하여 승인(B-2406-905-301)을 받은 후 수행하였다. 연구 대상자에게 연구 목적과 절차에 대해 충분히 설명하고 자발적으로 동의한 경우에만 동의서를 취득하였다. 연구 참여 도중 철회가 언제든지 가능하며 이에 따른 불이익이 없음과, 연구를 위해 수집되는 정보는 불필요한 개인식별자료는 제거한 채로 분석되며 연구 목적 이외에는 사용하지 않을 것임을 설명하였다. 수집된 정보는 생명윤리법에 의거하여 연구 종료 시점부터 3년 동안 안전하게 관리할 예정이며, 보관기관이 경과한 자료 중 개인정보와 관련된 부분은 개인정보보호법에 의거하여 파기할 예정이다.

## 연구 결과

### 1. 연구 대상자의 일반적 및 수술 관련 특성

본 연구에는 총 128명의 대상자가 참여하였으며, 이 중 남성이 90명(70.3%), 여성이 38명(29.7%)이었다. 대상자의 평균 연령은 65.63 ± 11.53세였으며, 모두 당뇨병 환자로 ASA class II에 해당하였다. 평균 체질량지수(body mass index)는 25.45 ± 3.69 kg/㎡였고, 평균 마취 시간은 120.24 ± 74.50분, 평균 수술 시간은 92.93 ± 69.72분이었다. 수술 진료과는 외과와 비뇨의학과가 각각 47명(36.7%)으로 가장 높은 비율을 차지하였으며, 마취 방법은 흡입 마취가 91명(71.1%)으로 가장 많았다. 수액 투여에 사용된 정맥 주사 바늘은 대부분 18 gauge로, 126명(98.4%)에서 사용되었다. 투여된 수액의 종류는 plasma solution이 85명(66.4%)으로 가장 많았으며, 총 수액 투여량은 평균 477.34 ± 276.36 mL였다(Table 1).

### 2. 수액 주입여부에 따른 양팔의 손가락 끝 혈당 수치

**Figure 1.**
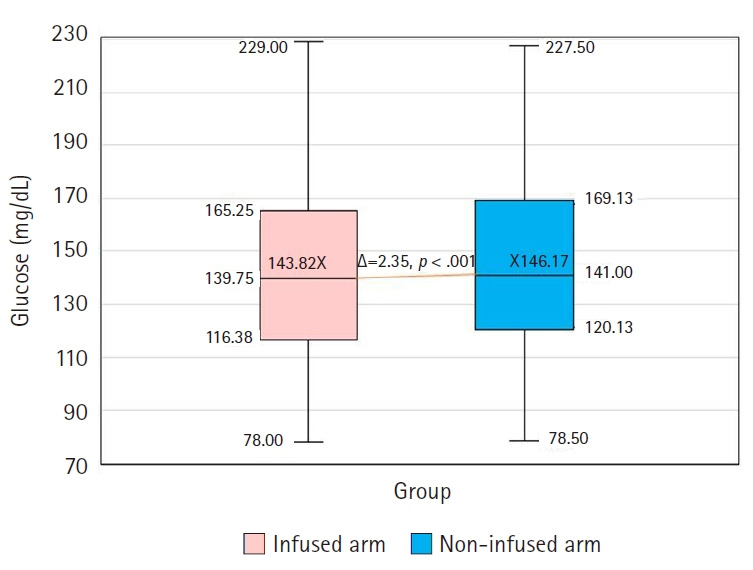
Comparison of fingertip blood glucose levels between intravenous fluid-infused and non-infused arms.

전신마취 하 수술 후 회복실에 입실한 당뇨병 환자를 대상으로 수액을 주입하고 있는 팔과 주입하지 않는 팔의 손가락 끝 혈당 수치의 평균에 유의한 차이가 있는 것으로 나타났다(t = −3.65, *p* < .001). 수액을 주입하고 있는 팔에서 측정한 혈당 수치는 평균 143.82 ± 36.44 mg/dL로 주입하지 않는 팔의 혈당 수치인 146.17 ± 37.46 mg/dL보다 낮았다(Table 2, Figure 1).

## 논의

본 연구에서 수액 요법을 적용하는 팔과 적용하지 않는 팔의 손가락 끝 혈당을 측정한 결과, 혈당 수치에 유의한 차이가 있었다. 수액을 주입하는 팔에서 측정한 혈당 수치는 143.82 ± 36.44 mg/dL이었고, 주입하지 않는 팔의 혈당 수치는 146.17 ± 37.46 mg/dL으로 수액이 주입되는 팔의 혈당 수치가 낮게 측정되었다. 이러한 결과는 모세 혈관에서 채혈할 때 피부 천자 부위 중 수액이 주입되는 손의 손가락은 반드시 피하라고 권고하는 크로아티아의 의학•생화학•실험의학협회 국가 가이드라인[24]에 부합한다.

하지만, 본 연구 결과는 포도당이 포함된 수액제제를 사용한 Park 등[6]의 연구에서 양 팔 간 혈당 수치 차이가 유의하지 않았던 결과와는 차이를 보인다. Park 등[6]의 연구는 당뇨병 대상자 수가 21명으로 적어 통계적 검정력이 제한되었을 가능성이 있으며, 포도당 수액을 사용함에 따라 포도당 자체의 영향이 혼재되어 있었던 반면, 본 연구는 128명의 당뇨병 환자를 대상으로 포도당이 포함되지 않은 정질용액을 사용함으로써 수액 주입 그 자체가 혈당에 미치는 영향을 보다 명확히 확인하였다는 점에서 의의가 있다.

일반적으로는 정맥 내 판막의 영향으로 혈류가 일정 방향으로 유지되지만[20], 혈액이 모세혈관을 따라 흐르면서 모세혈관 벽을 통해 서로 다른 물 분자와 이온들이 모든 방향으로 확산이 일어나기 때문에[25] 수액이 들어가는 팔의 손가락 끝 모세혈관에 수액의 영향이 없다고 단정하기는 어렵다고 사료된다. 특히, 혈당 측정기로 혈당을 측정하기 위해 채취하는 혈액은 진피의 모세혈관 혈액으로, 장치를 통해 피부를 찌르면 진피에서 절단된 세동맥과 정맥의 혈액이 모두 소량 섞여 혼합된 농도로 제공된다[26]. 또한, 정맥 내로 주입된 정질용액은 혈관 벽을 자유롭게 투과하여 약 25%는 혈관 내에 머물고 75%는 간질 조직으로 이동하는데[27], 많은 양의 정질용액이 정주되면 말초부종의 발생 가능성이 증가하고[28], 부종 시에는 모세혈관 위에 있는 부종 조직의 액체에 의해 혈액이 희석되어 측정된 혈당 농도를 인위적으로 낮출 수 있으므로 결과 해석 시 주의가 필요하다고 하였다[29]. 본 연구에서 환자에게 주입된 평균 수액량은 시간당 약 238.7 mL로 Park 등[6] 연구의 시간당 약 78.5 mL보다 많았고, 많은 양의 정질용액 주입으로 혈액이 희석되어 수액이 주입되는 팔과 주입되지 않은 팔의 혈당에 차이가 나타난 것으로 생각된다. 그러므로 수술을 받는 환자 중 혈당의 정확한 조절이 필요한 환자의 경우 잘못된 판독을 방지하기 위해 수액이 주입되는 팔의 손가락보다는 주입되지 않는 팔의 손가락을 선택하여 혈당 수치를 확인하는 것이 필요하겠다.

혈당의 섬세한 조절은 당뇨병 합병증을 줄이는 데 중요한 역할을 하며[30], 임상에서는 손가락 끝에서 측정한 혈당 수치를 근거로 고혈당 및 저혈당에 대한 처치 계획을 결정하기 때문에 가능한 한 정확한 혈당값을 얻는 것이 필수적이다[31]. 혈당 측정이 부정확할 경우 인슐린 또는 포도당 투여의 오류로 이어져[27] 혈당 조절에 부정적인 영향을 미치고, 환자에게 직접적인 위해를 줄 수 있으므로 정확한 혈당 측정은 매우 중요하다[32].

본 연구는 수액 요법 중인 당뇨병 환자의 혈당 수치가 측정 부위에 따라 달라질 수 있음을 확인하였고, 이에 따라 치료 계획 및 중재에도 차이가 발생할 수 있음을 시사하였다. 현재 임상 현장에서는 수액이 주입되는 팔을 혈당 측정 부위로 사용하는 것을 피하는 경향이 있으나, 이에 대한 명확한 근거는 크로아티아 의학•생화학•실험의학협회 국가 가이드라인[24] 외에는 찾을 수 없었다. 기존 연구 중 Park 등[6]은 포도당이 포함된 수액을 주입한 팔과 주입하지 않은 팔 사이 혈당 차이가 통계적으로 유의하지 않았다고 보고하였으나, 표본 수가 21명으로 본 연구의 128명에 비해 적고, 포도당 수액을 사용함에 따라 수액 자체가 혈당에 미친 영향을 배제하기 어려웠다는 한계가 있다. 이러한 점에서 본 연구는 비포도당성 정질용액을 사용하여 수액 주입 팔에서의 혈당이 희석되어 낮게 측정될 수 있다는 기존 가이드라인[24]의 권고를 실증적으로 검토하였다. 이러한 결과는 임상 간호 현장에서 수액 주입 팔을 혈당 측정 부위로 사용하는 것을 회피하는 현재의 실무적 판단에 과학적 근거를 제공한다는 점에서 의의가 있다.

특히, 혈당 측정은 당뇨병 간호 중재의 핵심 요소이며 임상 현장에서 빈번하게 수행되는 실무임에도 불구하고, 수액 요법 시 혈당 측정 부위에 따른 정확성에 관한 연구는 드문 실정이다. 따라서, 본 연구는 임상 간호 실무 개선에 기여할 수 있는 기초 자료로서 의미를 가진다.

다만, 본 연구는 단일 대학병원 마취회복실에 입실하여 정질용액을 주입받은 전신마취 수술 당뇨병 환자를 대상으로 하였으며, 혈당 측정 기기의 오차 범위 또한 존재하므로 연구 결과의 일반화에는 한계가 있다. 향후 연구 결과의 일반화를 위해 반복 연구가 필요하다.

## 결론

본 연구 결과 수액을 주입하고 있는 팔과 주입하지 않는 팔의 손가락 끝에서 측정한 혈당 수치의 평균에 차이가 있음을 확인하였다. 이를 통해, 임상 현장에서 수액이 주입되지 않는 팔의 손가락 끝에서 혈당을 측정하는 기존 간호 실무에 과학적 근거를 제공할 수 있었다. 이에 따라, 전신마취 수술 후 마취회복실에 입실한 당뇨병 환자의 혈당 측정 시에는 수액이 주입되지 않는 팔의 손가락 끝을 사용하는 것이 권장된다. 또한, 당뇨병 환자의 간호 중재에 필요한 정확한 혈당 측정 방법을 일반화하기 위해, 다양한 임상 환경을 반영하여 수액 종류, 연구 대상자, 연구 설계를 달리하여 후속 연구가 필요함을 제언한다.

## Figures and Tables

**Table 1.** Patient Demographics and Surgical Characteristics (N = 128)

Characteristics		n (%) or M ± SD
Sex	Men	90 (70.3)
	Women	38 (29.7)
Age (year)		65.63 ± 11.53
ASA class	Ⅱ	128 (100.0)
BMI (kg/㎡)		25.45 ± 3.69
Duration of anesthesia (minutes)		120.24 ± 74.50
Duration of operation (minutes)		92.93 ± 69.72
Department	General surgery	47 (36.7)
	Urology	47 (36.7)
	Neurosurgery	14 (10.9)
	Otorhinolaryngology	7 (5.5)
	Others	7 (5.5)
	Obstetrics and gynecology	6 (4.7)
Type of anesthesia	Inhalation general anesthesia	91 (71.1)
	Intravenous general anesthesia	37 (28.9)
Type of IV catheter gauge	18 G	126 (98.4)
	20 G	2 (1.6)
Type of fluid	Plasma solution	85 (66.4)
	Normal saline	33 (25.8)
	Hartmann’s solution	10 (7.8)
Total amount of fluid (mL)		477.34 ± 276.36

M = Mean; SD = Standard deviation; ASA = American Society of Anesthesiologists; BMI = Body mass index; IV = Intravenous; G = Gauge.

**Table 2.** Comparison of Fingertip Blood Glucose Levels between Intravenous Fluid-infused and Non-infused Arms (N = 128)

Measured arm	First blood glucose (mg/dL)	Second blood glucose (mg/dL)	Average blood glucose (mg/dL)	t	*p*
Infused arm	142.18 ± 35.78	145.45 ± 37.41	143.82 ± 36.44	−3.65	< .001
Non-infused arm	144.79 ± 37.50	147.56 ± 75.42	146.17 ± 37.46		

Values are presented as the mean ± standard deviation.

## Data Availability

Please contact the corresponding author for data availability.
